# Physicochemical and biological evaluation of JR-131 as a biosimilar to a long-acting erythropoiesis-stimulating agent darbepoetin alfa

**DOI:** 10.1371/journal.pone.0231830

**Published:** 2020-04-17

**Authors:** Junya Tani, Yae Ito, Satoshi Tatemichi, Makoto Yamakami, Tsuyoshi Fukui, Yukichi Hatano, Shinji Kakimoto, Ayaka Kotani, Atsushi Sugimura, Kazutoshi Mihara, Ryuji Yamamoto, Noboru Tanaka, Kohtaro Minami, Kenichi Takahashi, Tohru Hirato

**Affiliations:** 1 Research Division, JCR Pharmaceuticals Co., Ltd., Kobe, Japan; 2 Research Division, Kissei Pharmaceutical Co., Ltd., Azumino, Japan; 3 Research Planning Division, JCR Pharmaceuticals Co., Ltd., Kobe, Japan; Universitatsklinikum Wurzburg, GERMANY

## Abstract

Renal anemia is predominantly caused by a relative deficiency in erythropoietin (EPO). Conventional treatment for renal anemia includes the use of recombinant human EPO (rhEPO) or a long-acting erythropoiesis-activating agent named darbepoetin alfa, which is a modified rhEPO with a carbohydrate chain structure that differs from native hEPO. We have developed a biosimilar to darbepoetin alfa designated JR-131. Here, we comprehensively compare the physicochemical and biological characteristics of JR-131 to darbepoetin alfa. JR-131 demonstrated similar protein structure to the originator, darbepoetin alfa, by peptide mapping and circular dichroism spectroscopy. Additionally, mass spectroscopic analyses and capillary zone electrophoresis revealed similar glycosylation patterns between the two products. Human bone marrow-derived erythroblasts differentiated and proliferated to form colonies with JR-131 to a similar degree as darbepoetin alfa. Finally, JR-131 stimulated erythropoiesis and improved anemia in rats similarly to darbepoetin alfa. Our data show the similarity in physicochemical and biological properties of JR-131 to those of darbepoetin alfa, and JR-131 therefore represents a biosimilar for use in the treatment of renal anemia.

## Introduction

Renal anemia is a characteristic complication of chronic kidney disease, which is caused predominantly by a relative deficiency in erythropoietin (EPO), an erythropoietic hormone that induces red blood cell production [1], which results in disabling fatigue, palpitations, shortness of breath, and cardiac failure in the end-stage of the disease. Until the end of the 1980s, correction of renal anemia was dependent on blood transfusions. However, the development of recombinant human EPO (rhEPO) has revolutionized the management of renal anemia [2].

EPO is a glycoprotein hormone produced by interstitial fibroblasts in the kidney and released into the blood in response to low blood oxygen levels [3]. EPO receptor (EPOR) mediates the erythropoietic function of EPO [4]. The highest expression level of EPOR is observed in late-stage erythroid progenitor cells [5, 6]. Activation of EPO-EPOR signaling promotes survival and prevents apoptosis of these progenitor cells, leading to their proliferation and differentiation to erythroblasts [7].

rhEPO was first introduced to international markets in 1989 as epoetin alfa, and many clinically approved rhEPO preparations have been commercially produced thereafter [8, 9]. Although rhEPO is highly effective in treating anemia and improving the quality of life of patients, receiving the drug two to three times per week at the clinic can be burdensome. From this point of view, a long-acting erythropoiesis-stimulating agent (ESA) darbepoetin alfa, was developed and approved in several countries for the treatment of renal anemia [10]. Darbepoetin alfa is a modified rhEPO with a different carbohydrate chain structure, which is created by replacing five amino acid residues of the native hEPO. Several candidate biosimilars to darbepoetin alfa are currently under development [11].

Biosimilars are biological drugs demonstrating highly similar safety, efficacy, and quality to already approved biotechnology-applied products, which may be less expensive than their reference medical product (RMP), and thus could save healthcare costs. Sponsors of biosimilar medicines are required to demonstrate that the product has comparable safety and efficacy to the RMP through comprehensive physicochemical and biological analyses, in addition to non-clinical and clinical data [12–14].

In the present study, we sought to demonstrate biosimilarity of JR-131, which has been developed by JCR Pharmaceuticals and Kissei Pharmaceutical, to its RMP darbepoetin alfa (NESP^®^, Kyowa Kirin, Tokyo, Japan). JR-131 is a recombinant human protein produced using Chinese hamster ovary (CHO) cells as a host cell line. According to the current regulatory authority guidelines [12, 13], we have analyzed the structural and functional similarities of JR-131 to those of its RMP. This study aims to assess the physicochemical and biological similarity between JR-131 and darbepoetin alfa.

## Materials and methods

### Materials

The biosimilar product, JR-131, was manufactured by JCR Pharmaceuticals. JR-131 was produced from an established CHO cell line without use of any other animal-derived raw materials. JR-131 is a glycoprotein, which contains 165 amino acids (C_800_H_1300_N_228_O_244_S_5_: the calculated molecular weight is 18,176.59) with five amino acid mutations relative to native hEPO (S1 Fig), similar to the RMP darbepoetin alfa (NESP^®^). In addition to three *N*-linked and one *O*-linked oligosaccharide that are found in native hEPO, JR-131 and darbepoetin alfa have two additional *N*-linked oligosaccharides (S1 Fig). A total of seven lots of JR-131 and six lots of darbepoetin alfa were analyzed in the present study, and the data presented are the results from representative lots unless otherwise indicated.

### Peptide mapping

Test substances were denatured with guanidine-HCl, reduced with dithiothreitol, and then alkylated with iodoacetic acid. The samples were subjected to size-exclusion chromatography using Sephadex^™^ G-25 superfine (5 mm ID and 150 mm in length, GE Healthcare, Buckinghamshire, UK) to purify the protein fraction, and then digested with endoproteinase Lys-C (Lys-C, Roche Diagnostics, Basel, Switzerland) at a 1:100 enzyme-to-protein ratio at 37°C for 3 h, and subsequently with 100 units of N-glycosidase (Roche Diagnostics)/mg protein at 37°C for 2 h. The digested peptides were separated by reverse-phase high performance liquid chromatography (RP-HPLC) using Proteonavi (4.6 mm ID and 150 mm in length, Osaka Soda, Osaka, Japan) at 40°C with a linear 2-propanol gradient containing 0.1% trifluoroacetic acid (TFA) at a flow rate of 1.0 mL/min. Absorbance was monitored at 215 nm.

### Circular dichroism (CD)

CD spectra were obtained with a JASCO J-802 spectropolarimeter (JASCO, Tokyo, Japan), as described previously [14]. Briefly, each sample was diluted to 0.2 mg/mL (far-UV) or 0.5 mg/mL (near-UV) with the JR-131 formulation buffer. The wavelength scan measurements were conducted at room temperature using 1 mm and 10 mm path-length cuvettes for far-UV (200 to 250 nm) and near-UV (250 to 320 nm), respectively. Data are expressed as molar ellipticities, calculated using a mean residue weight for the protein moiety.

### Identification of glycosylation sites

Test substances were denatured, reduced, and alkylated using a procedure similar to that used for peptide mapping. The samples were purified, vacuum-dried, and dissolved in Tris-acetate buffer (pH 8.5) and digested with trypsin at 37°C for 18 h. The tryptic digests were mixed with Sepharose CL-4B in a 1-butanol/ethanol/H_2_O solution (4:1:1). After washing twice with the solution, the gels were incubated in ethanol/H_2_O solution (1:1). Solution phases were recovered as glycopeptide fractions. The tryptic digests and the glycopeptide fractions were then separated by RP-HPLC using a YMC-Pack C8 column (4.6 mm ID and 150 mm in length, YMC, Kyoto, Japan) at 25°C with a linear gradient of acetonitrile containing 0.06% TFA. Peak fractions derived from the glycopeptides were subjected to amino acid sequencing using a protein sequencer (PPSQ-33A, Shimadzu, Kyoto, Japan).

### Monosaccharide analysis

Test substances were desalted using ultrafiltration via an ultrafiltration filter unit, and 20 μg aliquots were hydrolyzed with 2.5 mol/L trifluoroacetic acid at 100°C for 3 h. The samples were then cooled, dried, and reconstituted in L-rhamnose solution. Neutral monosaccharide was analyzed using a Shin-pack ISA-07/S2504 column (0.7 μm ID and 250 mm in length, Shimadzu) with a 50-min linear gradient of 0.1–0.4 mol/L potassium buffer (pH 8.0–9.0) by the Reducing Sugar Analysis System (Shimadzu). For amino sugar determination, test substances were hydrolyzed with 4 mol/L HCl at 100°C for 3 h, cooled, dried, and reconstituted in 0.02 mol/L HCl. An amino acid analyzer JLC-500/V2 (JEOL, Tokyo, Japan) was used for the analysis. For sialic acid analysis, test substances were treated with 0.1 mol/L NaOH at 37°C for 30 min, neutralized with HCl, and then reacted with 0.035 mol/L HCl at 70°C for 140 min to release sialic acids. The released sialic acids were fluorescently labeled with 1,2-diamino-4,5-methylenedioxybenzene at 50°C for 150 min. The solutions were analyzed by RP-HPLC using a COSMOSIL 5C18-PAQ column (4.6 mm ID and 150 mm in length, Nacalai Tesque, Kyoto, Japan) with a methanol/acetonitrile/water solvent system and fluorescence detection (Ex: 373 nm, Em: 448 nm). Quantification was performed relative to the appropriate monosaccharide standard curve.

### *N*-linked oligosaccharide profiling

Test substances were denatured, reduced, alkylated, and purified with a procedure similar to that used for peptide mapping. The samples were digested with 100 units of N-glycosidase (Roche Diagnostics)/mg protein at 37°C for 48 h, and the supernatants after ethanol precipitation were vacuum-dried. The samples were then dissolved in 0.1 mol/L NaOH and separated by high performance anion exchange chromatography with pulsed amperometric detection (HPAEC-PAD, Thermo Fisher Scientific, Waltham, MA) using a CarboPac PA1 column (4 mm ID and 250 mm in length, Thermo Fisher Scientific) at 25°C with a linear gradient of 0.03–0.20 mol/L sodium acetate in 0.1 mol/L NaOH for 100 min at a flow rate of 1.0 mL/min.

### *O*-linked oligosaccharide profiling

Test substances were pretreated with a BlotGlyco BS-45450 kit (Sumitomo Bakelite, Tokyo, Japan) according to the manufacturer’s instructions. Briefly, desalted test substances were mixed with the *O*-glycan releasing agent included in the kit, heated at 55°C for 5 h and vacuum-dried at 60°C for more than 16 h. The samples were dissolved in H_2_O, added to BlotGlyco beads, and immobilized with acetonitrile/acetic acid (49:1). After washing the beads, the reduced ends of the purified glycans were labeled with 2-aminobenzamide (2-AB, Alfa Aesar, Lancashire, UK). These samples were analyzed using an ACQUITY UPLC Glycan BEH Amide Column (2.1 mm ID and 150 mm in length, Waters, Milford, MA) at 45°C with an ammonium formate/acetonitrile gradient at a flow rate of 0.25 mL/min for 35 min. The fluorescence was monitored at 330 nm for excitation and 420 nm for emission.

### Mass spectrometric profiling of glycopeptides

Test substances were denatured, reduced, alkylated, and purified with a similar procedure to that used for peptide mapping. The samples were digested with trypsin at 37°C for 20 h and then with Endoproteinase Glu-C at 25°C for 18 h. These samples were analyzed by liquid chromatography-mass spectrometry (LC/MS) using the Vanquish UHPLC System (Thermo Fisher Scientific) equipped with a Cadenza CD-C18 column (2 mm ID and 150 mm in length, Imtakt, Kyoto, Japan) and Q Exactive™ Plus (Thermo Fisher Scientific). The scan range was *m/z* 500–2500 for positive ion mode analysis. The mass spectra were deconvoluted to specify molar masses (BioPharma Finder 2.0, Thermo Fisher Scientific) and the glycopeptides were identified.

### Capillary zone electrophoresis (CZE)

Desalted test substances were filtered using a 0.45 μm filter (Merck, Darmstadt, Germany) and analyzed with the PA800s plus capillary electrophoresis system (Beckman Coulter, Fullerton, CA) equipped with a fused silica capillary (50 μm ID and ~40 cm in length; AB Sciex, Framingham, MA). The samples were injected at 0.5 psi for 20 s and then separated at 20.0 μA at 35°C for 30 min. The CZE buffer (pH 4.5) used in this experiment contained 1,4-diaminobutane, sodium acetate, and urea. The absorbance was monitored at 214 nm.

### Matrix-assisted laser desorption ionization time-of-flight mass spectrometry (MALDI-TOF/MS) analysis

The molecular weights of JR-131 and darbepoetin alfa were analyzed by MALDI-TOF/MS using the microflex LRF (Bruker, Billerica, MA). Desalted test substances (2 μL at approximately 0.5 mg/mL) were mixed with an equal volume of 20 mg/mL 2,5-dihydroxybenzoic acid dissolved in 0.1% trifluoroacetic acid/acetonitrile (7:3) (matrix solution) and then air-dried. The samples were analyzed by MALDI-TOF/MS in positive ion linear mode. Calibration was performed using the Protein Standard II (Bruker) protein mixture.

### Immunoblotting

Test substances (0.1 μg/lane) were separated by sodium dodecyl sulfate-polyacrylamide gel electrophoresis (SDS-PAGE) under reducing conditions and transferred to a polyvinylidene difluoride membrane (Bio-Rad Laboratories, Hercules, CA) using the Trans-Blot^®^ SD Semi-Dry Transfer Cell (Bio-Rad Laboratories). After blocking with Tris-buffered saline (TBS) containing 5% skim milk, the membrane was immersed in the primary antibody solution (0.1% bovine serum albumin/0.05% Tween-20/TBS) containing anti-human EPO monoclonal antibody (clone AE7A5, R&D Systems, Minneapolis, MN) for 1 h with gentle rocking. Then, the washed membrane was immersed in a secondary antibody solution containing alkaline phosphatase-labeled goat anti-mouse IgG antibody (Bio-Rad Laboratories) for 1 h with gentle shaking. Subsequently, the separated proteins were visualized with a BCIP/NBT Color Development Substrate (Promega, Madison, WI).

### Receptor binding affinity

The EPOR binding affinity of the test substances was evaluated using the Biacore T100 surface plasmon resonance system (GE Healthcare). Recombinant hEPOR-Fc chimera protein (hEPOR-Fc) was fixed on Sensor Chip Protein A (GE Healthcare), and solutions of JR-131 or darbepoetin alfa (at final concentrations of 0.25–4.00 nmol/L) were reacted with the immobilized hEPOR-Fc at a flow rate of 90 μL/min using a single-cycle kinetics approach. The contact time and dissociation time were set at 90 sec and 3,000 sec, respectively. The equilibrium dissociation constant (K_D_) was calculated from the sensorgram.

### *In vitro* efficacy assays

The agonist effect of the test substances was comparatively examined using cell growth assays with BaF/EPOR cells, which express human EPOR and show hEPO-induced proliferation in a concentration-dependent manner. The BaF/EPOR cells were seeded at 5 × 10^3^ cells/well in a 96-well plate, and JR-131 or darbepoetin alfa (final concentration: 0.005–5 ng/mL) was added. After 48 h of culture at 37°C, cell growth was assessed based on the degree of reduction of the MTS tetrazolium compound to formazan product using the CellTiter 96^®^ Aqueous One Solution Cell Proliferation Assay (Promega).

The effects of the test substances on colony-forming unit-erythroid (CFU-E)- and burst-forming unit-erythroid (BFU-E)-derived colony formation in human bone-marrow-derived mononuclear cells (purchased from AllCells, CA) were examined. The cells were obtained according to collection protocols and donor informed consent, which were approved by an Institutional Review Board, with strict oversight (https://www.allcells.com/cell-tissue-procurement/donor-facilities/). JR-131 or darbepoetin alfa was diluted with StemSpan^®^ SFEM (StemCell Technologies, Seattle, WA) to concentrations of 1.0; 10; 100; 1,000; 10,000; and 100,000 ng/mL. The cells were suspended at a concentration of 3.38 × 10^5^ cells/mL. Each diluted sample (44 μL) and 356 μL of cell suspension were added to 4 mL of MethoCult^®^ SF H4236 (StemCell Technologies) containing 5.5 ng/mL recombinant human stem cell factor (rhSCF, PeproTech, Rocky Hill, NJ). Three aliquots of 1.1 mL of the mixed cell suspensions were seeded on 35-mm culture dishes and cultured for 13–14 days at 37°C under 5% CO_2_. At the end of the culture period, the numbers of CFU-E- and BFU-E-derived colonies were counted under a phase contrast microscope.

### *In vivo* efficacy assay using nephrectomized rats

As an animal model of renal anemia, male Sprague-Dawley rats who had undergone 5/6 nephrectomy were purchased from Japan SLC (Hamamatsu, Japan). Two-thirds of their left kidneys had been surgically resected at 8 weeks of age and right uninephrectomy had been performed one week later. A total of 68 rats (56 for nephrectomized and 12 for sham-operated) were purchased. The sham-operated rats were used as normal controls to confirm successful nephrectomy-induced renal failure in the other rats. All rats were housed in wire cages with free access to normal chow and tap water in a room with controlled temperature (22 ± 3°C) and humidity (50 ± 20%), and a 12-h light/dark cycle. The animals were assigned to groups given JR-131, darbepoetin alfa (0.3 or 1 μg/kg, intravenously once weekly for 4 weeks), or vehicle (n = 10 for each group) so as to minimize the variation in their hemoglobin concentration, reticulocyte number, serum urea nitrogen, creatinine, and body weight. Administration of the test substances via tail vein injection was initiated at 11 weeks of age. During the test period, blood samples were obtained from the jugular vein at the indicated time points to measure hemoglobin concentration and reticulocyte number. One to three animals in each group died probably due to renal failure. The number of animals used for data analysis is indicated in each corresponding figure. After completion of the study, the rats were euthanized by CO_2_ exposure. These experiments were performed at the Bioresearch Center, CMIC Pharma Science (Yamanashi, Japan), which is certified by the Association for Assessment and Accreditation of Laboratory Animal Care International, with the approval of the Institutional Animal Care and Use Committee of CMIC Pharma Science (approved number: 2016–99).

## Results

### Protein structure

**Fig 1:**
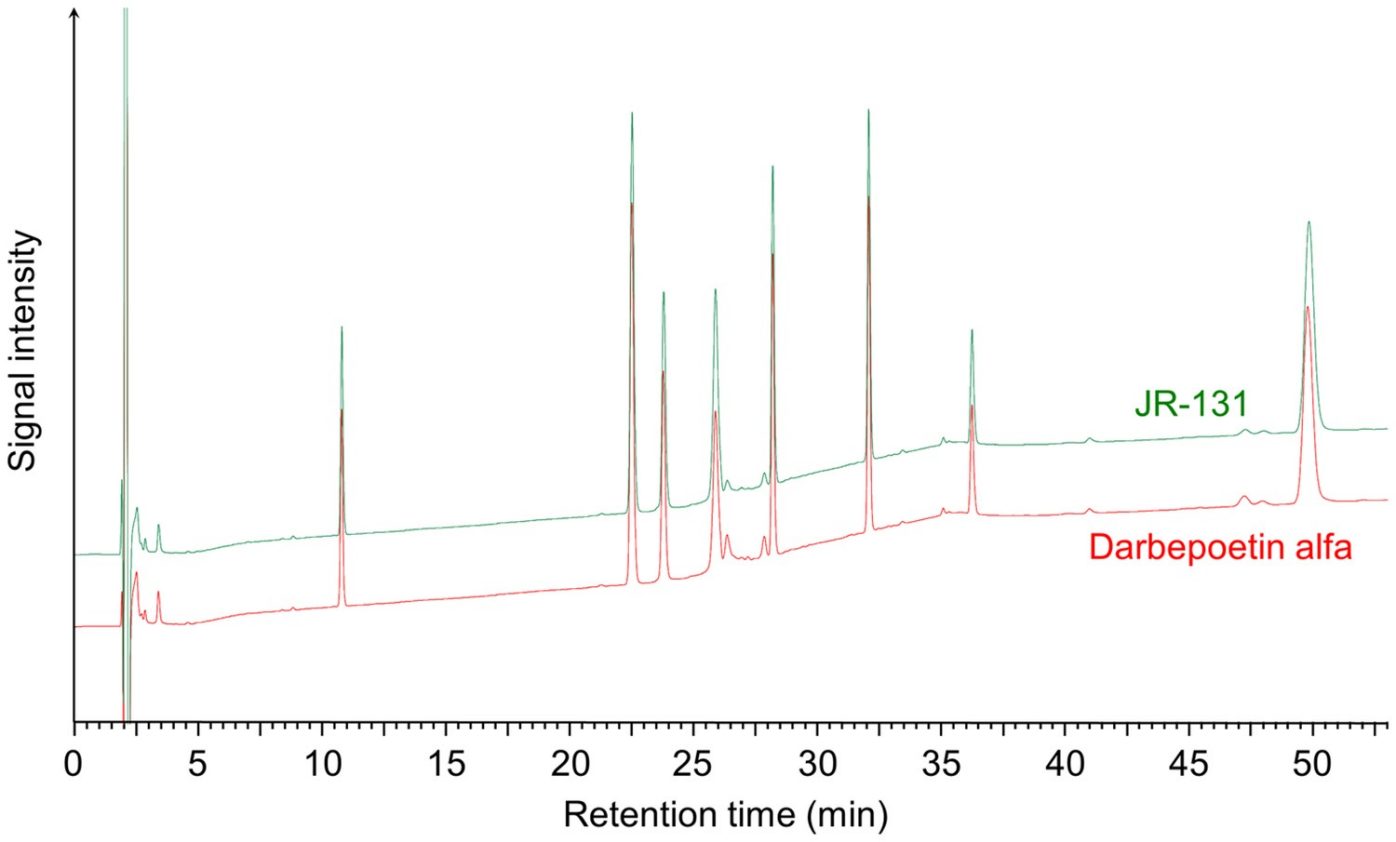
Peptide mapping profiles of JR-131 and darbepoetin alfa. Representative profiles of JR-131 (green) and darbepoetin alfa (red) are presented.

We first compared the primary and higher-order structures of JR-131 to those of its originator, darbepoetin alfa. The N-terminal 20 amino acid sequence of JR-131 determined by Edman degradation was the same as that of darbepoetin alfa (N-APPRLIXDSRVLERYLLEAK, where X was not detected due to disulfide bond formation between cysteine amino acids). C-terminal analysis of Lys-C-digests by HPLC revealed that the peptide lacking the C-terminal Arg166 was a major molecular species (> 99%) in both JR-131 and darbepoetin alfa. For peptide mapping, Lys-C- and N-glycosidase-digested peptides were subjected to HPLC analysis. As darbepoetin alfa has eight Lys residues, nine peptide fragments should have been generated by Lys-C digestion, in theory. However, one of the fragments could not be detected by the HPLC analysis because it consisted of only two amino acids. The peptide mapping chromatograms contained eight major peaks that were similar between JR-131 and darbepoetin alfa (Fig 1). In addition, the complete sequences of the two products were determined to be identical. These results demonstrate that the primary structures of JR-131 and darbepoetin alfa are identical.

**Fig 2:**
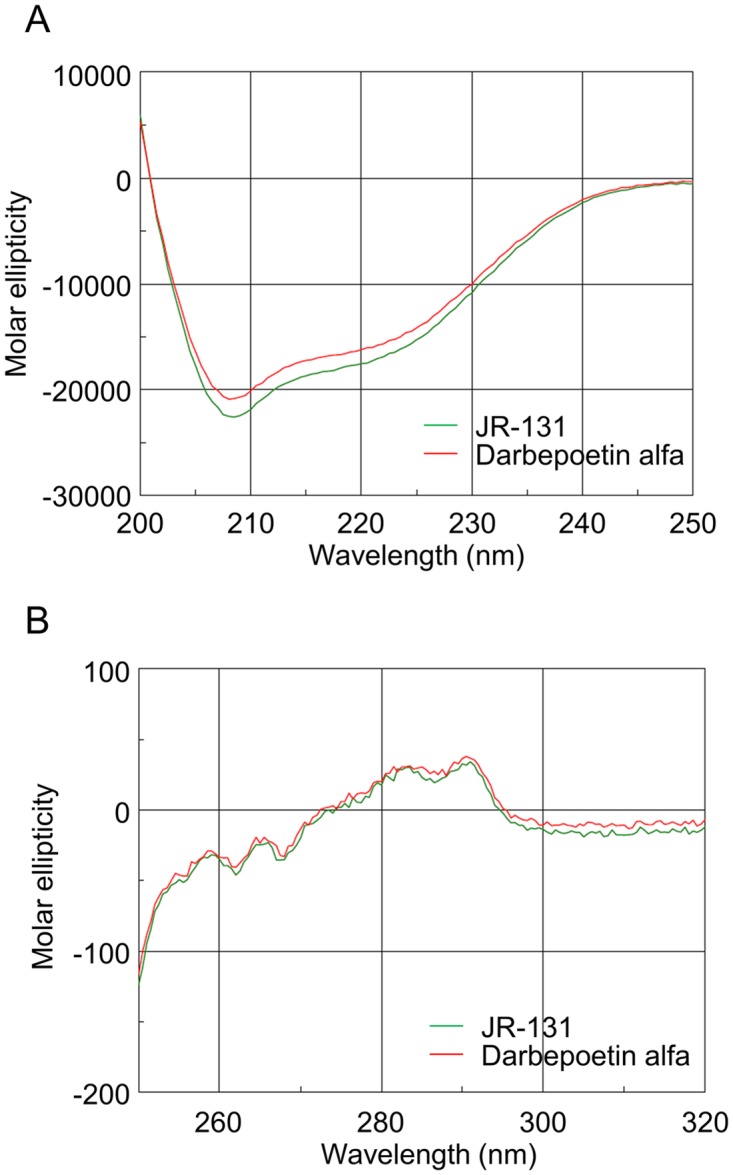
CD spectra of JR-131 and darbepoetin alfa. (A) Far-UV and (B) near-UV spectra are presented.

Higher-order structures of the test substances were determined using CD spectroscopy. The far-UV CD spectrum of JR-131 was nearly identical to that of darbepoetin alfa (Fig 2A). Comparatively, the near-UV spectra of the two substances were shown to overlap with each other (Fig 2B). The secondary protein structures were analyzed using far-UV CD spectroscopy, the results of which are summarized in Table 1, further demonstrating similarities between JR-131 and darbepoetin alfa. These results indicate that there are no substantial differences in the higher-order structures of JR-131 and darbepoetin alfa.

**Table 1:** Analysis of the secondary structure of JR-131 and darbepoetin alfa from CD spectra.

Test substance	α-helix	β-sheet	β-turn	Random coil
JR-131 lot #1	35%	25%	11%	30%
JR-131 lot #2	35%	23%	13%	29%
Darbepoetin alfa	36%	24%	12%	29%

### Glycosylation

Protein sequencing of trypsin-digested samples and glycopeptides showed that amino acid residues 24, 30, 38, 83, 88 (all Asn residues, deduced from cDNA), and 126 (Ser residue) were undetectable. This suggests that JR-131 has five potential *N*-linked oligosaccharide binding sites (Asn24, Asn30, Asn38, Asn83, and Asn88) and one potential *O*-linked oligosaccharide binding site (Ser126) as previously shown for darbepoetin alfa [15]. Moreover, the sugar composition was similar between JR-131 and darbepoetin alfa (Table 2).

**Table 2:** Sugar composition of JR-131 and darbepoetin alfa.

Sugar composition	Content (mol/mol)	
	JR-131	Darbepoetin alfa
N-acetylneuraminic acid	19	18
N-glycolylneuraminic acid	0.07	0.05
Mannose	9.9	10.2
Fucose	4.2	4.3
Galactose	17.0	16.8
Glucosamine	25.8	24.8
Galactosamine	0.80	0.76

**Fig 3:**
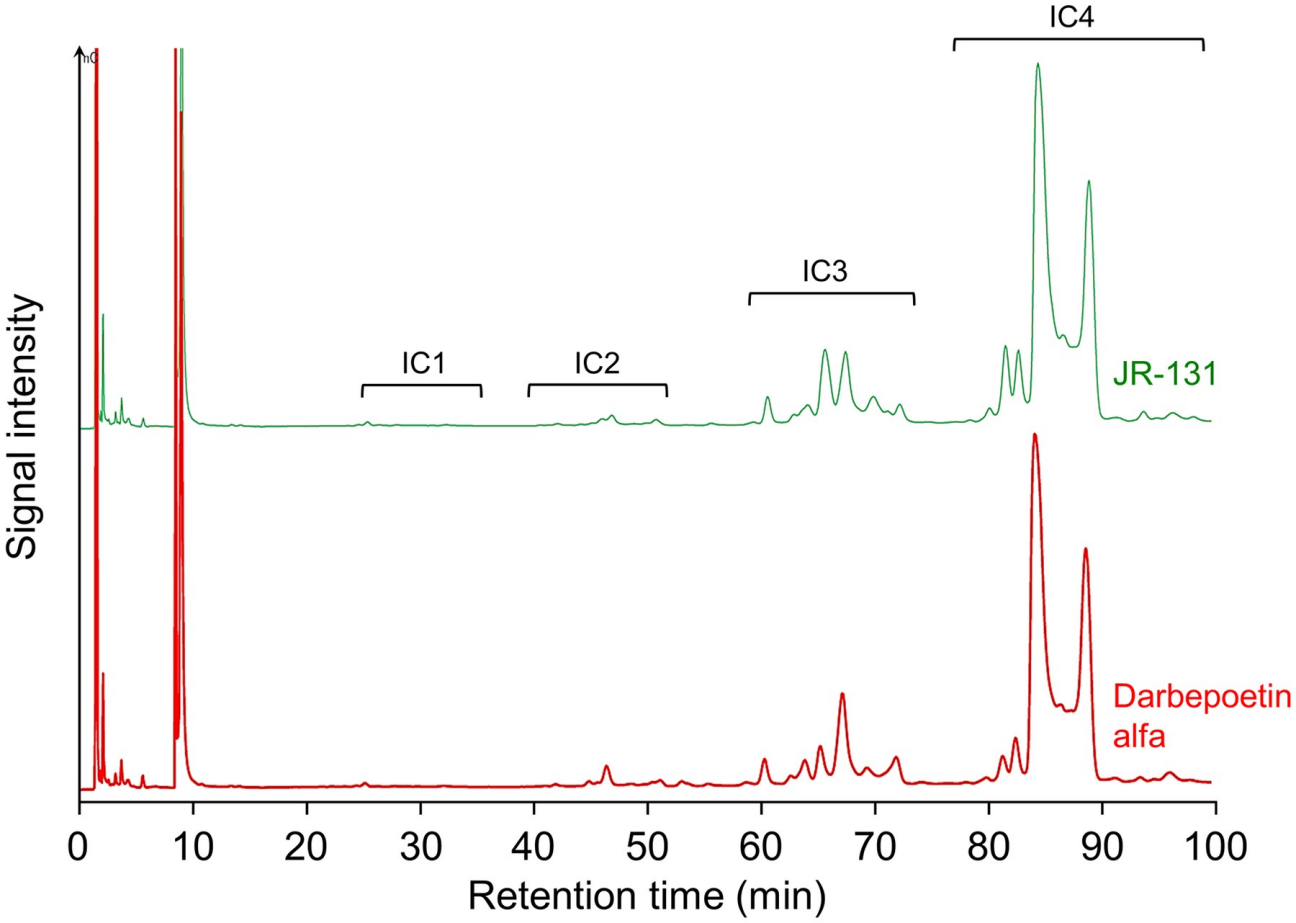
*N*-linked oligosaccharide profiles of JR-131 and darbepoetin alfa. IC1, IC2, IC3, and IC4 represent monosialo, disialo, trisialo, and tetrasialo sugar chain peak groups, respectively.

**Fig 4:**
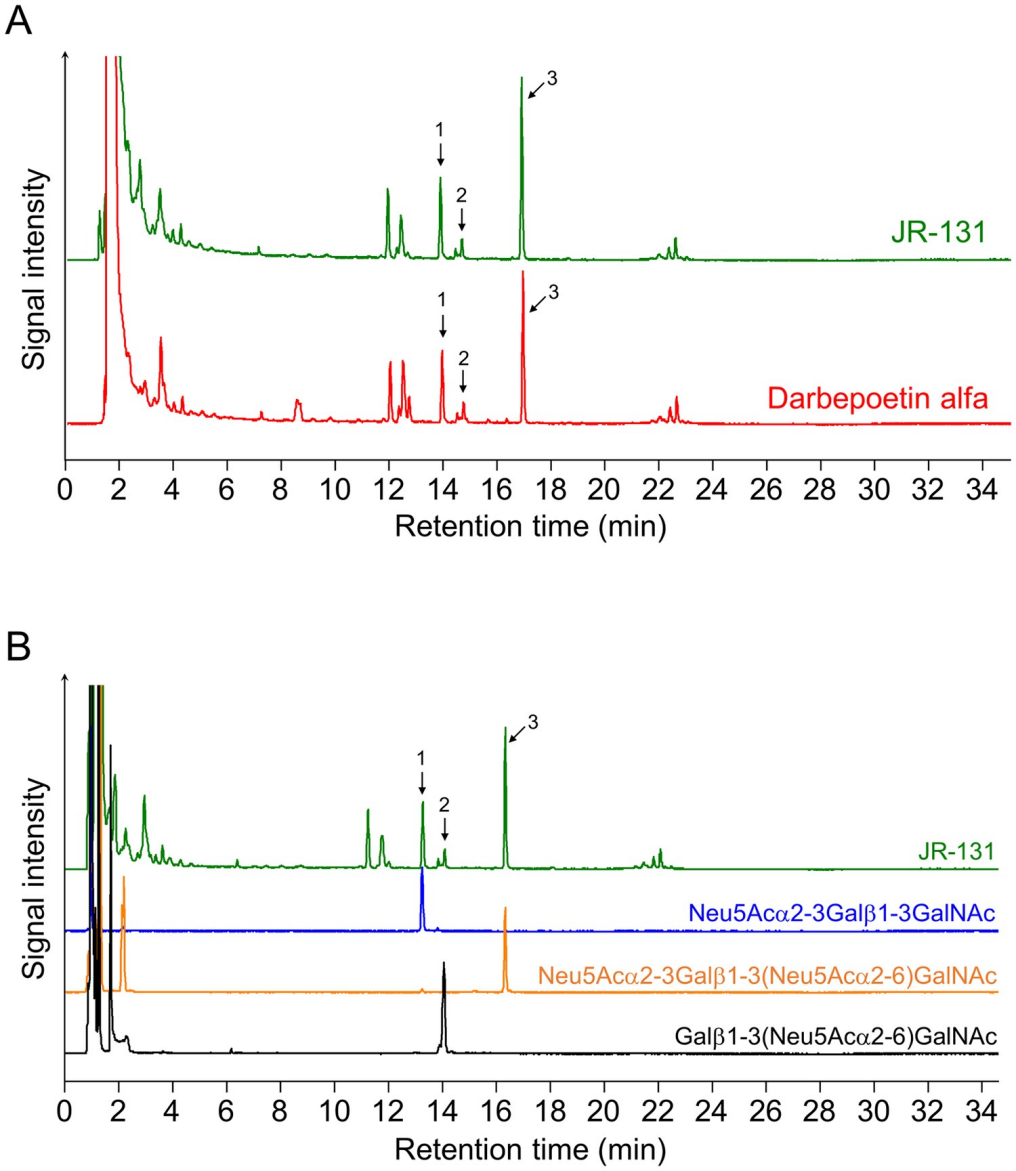
*O*-linked oligosaccharide profiles of JR-131 and darbepoetin alfa. (A) Overlaid chromatogram of *O*-linked oligosaccharide profiles of JR-131 and darbepoetin alfa. (B) Determination of the peaks. Galβ1-3(Neu5Acα2–6)GalNAc was obtained by treatment of the standard glycan (2AB-labeled di-sialylated core 10 glycan) with α-2,3 sialidase.

The *N*-linked oligosaccharide profiles are shown in Fig 3. Four peak groups, IC1, IC2, IC3, and IC4 represent monosialo, disialo, trisialo, and tetrasialo sugar chains, respectively. The *N*-linked oligosaccharide profile of JR-131 was similar to that of darbepoetin alfa (Fig 3). The *O*-linked oligosaccharide profile of JR-131 is also similar to that of the originator (Fig 4A). Since peaks 1 and 3, and the 2-AB-labeled standard glycans (2AB-labeled sialylated core 10 glycan and 2AB-labeled disialylated core 10 glycan, Ludger, Oxfordshire, UK) had the same retention times (Fig 4B), their molecular structures were determined to be Neu5Acα2-3Galβ1-3GalNAc and Neu5Acα2-3Galβ1-3(Neu5Acα2–6)GalNAc, respectively. The retention time of peak 2 was the same as that of the α-2,3 sialidase-treated 2AB-labeled disialylated core 10 glycan (Fig 4B). Thus, the molecular structure of peak 2 was determined to be Galβ1-3(Neu5Acα2–6)GalNAc.

**Fig 5:**
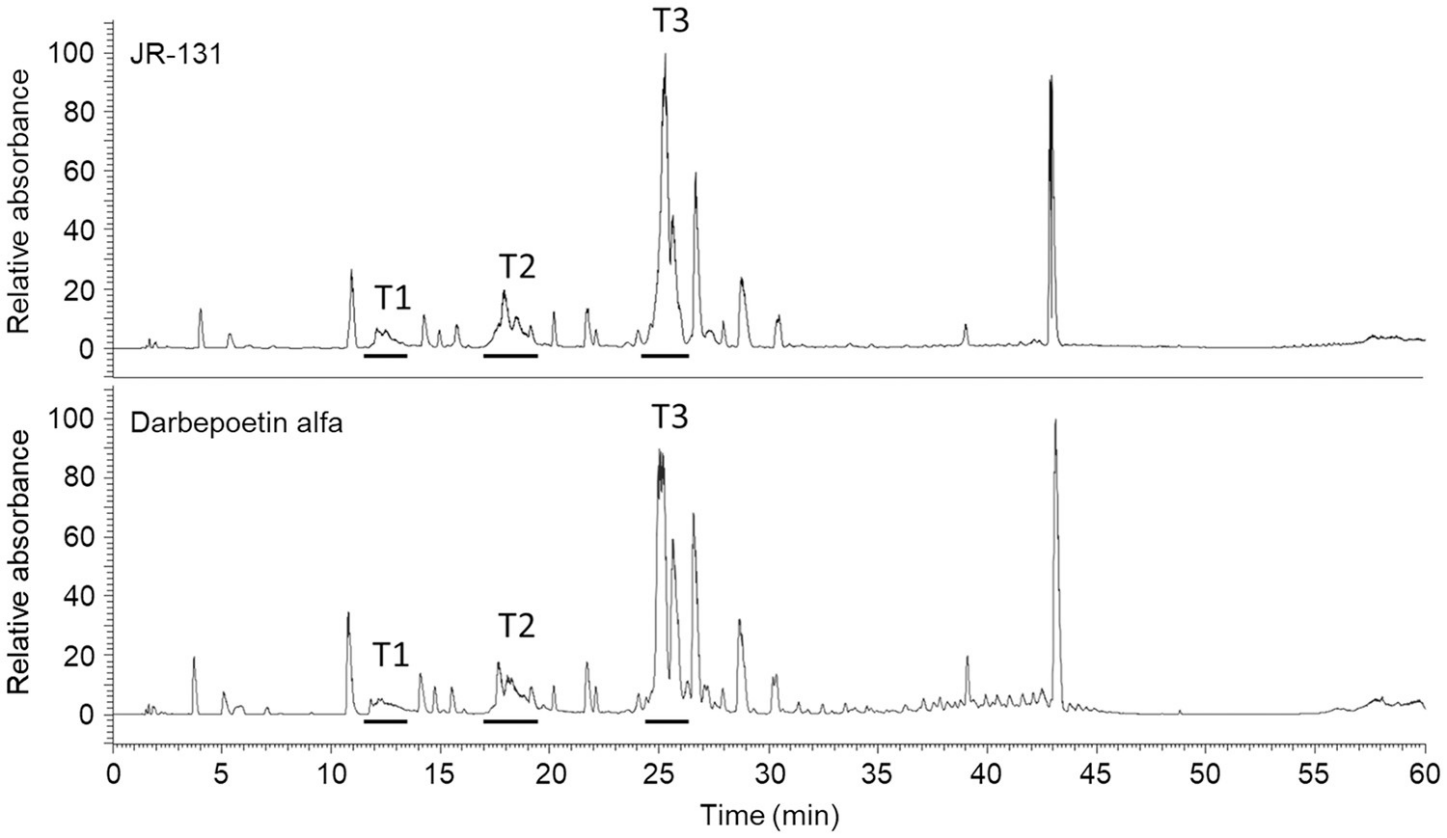
Total ion chromatograms of trypsin/endoproteinase Glu-C-digested JR-131 (upper) and darbepoetin alfa (lower). Fractions from retention times 11.5 to 13.5 min (T1), 17.0 to 19.5 min (T2), and 24.0 to 26.5 min (T3) were subjected to mass spectrometric profiling (S2A Fig for T1, S2B Fig for T2, and S2C Fig for T3).

Next, glycopeptide analysis was performed to compare glycan variations between JR-131 and darbepoetin alfa. Trypsin/endoproteinase Glu-C-digests were separated by RP-HPLC (Fig 5). The mass spectra from retention times 11.5 to 13.5 min (T1), 17.0 to 19.5 min (T2), and 24.0 to 26.5 min (T3) were deconvoluted to specify molar masses (S2A Fig for T1, S2B Fig for T2, and S2C Fig for T3). Each glycopeptide derived from the two substances was detected at the same retention time (T1, T2, and T3 in Fig 5), and their respective mass spectra were similar (S2A–S2C Fig). Signals corresponding to peptide Glu21-Glu31 with two sugar chains and peptide Asn24-Glu31 with one sugar chain were detected in T1 (S2A Fig). Signals corresponding to peptide Gly77-Glu89 with two sugar chains and peptide Asn38-Lys45 with one sugar chain were detected in T2 (S2B Fig). A signal corresponding to peptide Thr32-Lys45 with one sugar chain was detected in T3 (S2C Fig). The dominant glycan compositions contain sialylated tetra-antennary structures (Gal_4_GlcNAc_4_Fuc_1_NeuAc_4_Man_3_GlcNAc_2_) at all glycosylation sites. Although the major peaks were not different between JR-131 and the originator, the signal intensities of the *O*-acetylated sialic acid ion-derived subpeaks were somewhat different (S2A–S2C Fig), which indicates that JR-131 contains less *O*-acetylated sialic acid than darbepoetin alfa and this may affect the therapeutic half-life of these drugs [16].

**Fig 6:**
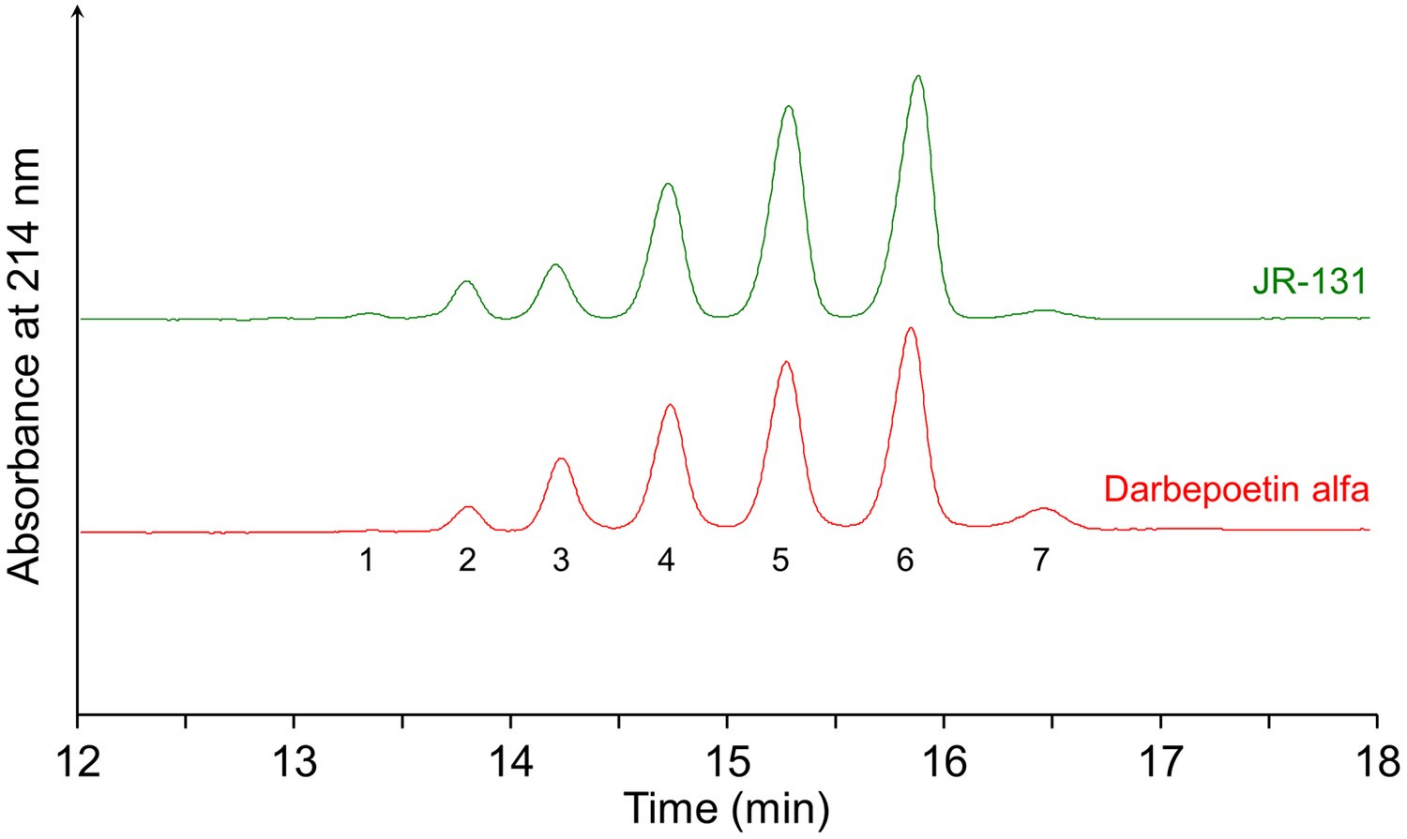
Capillary zone electropherograms of JR-131 and darbepoetin alfa. Charged isomers were labeled with peak numbers 1 to 7 in the direction from basic to acidic regions.

Differences in the number of terminal sialic acids account for the presence of several charged isomers of darbepoetin alfa [9]. Since isomer distribution may affect drug efficacy, CZE was performed to separate and quantify the charged isomers. Seven peaks were detected, and the ratio of each peak area to the total area was not different between the two products (Fig 6).

Collectively, all of the glycosylation analyses described above indicate that JR-131 has a highly similar sugar chain structure to that of darbepoetin alfa.

### Molecular weight

**Fig 7:**
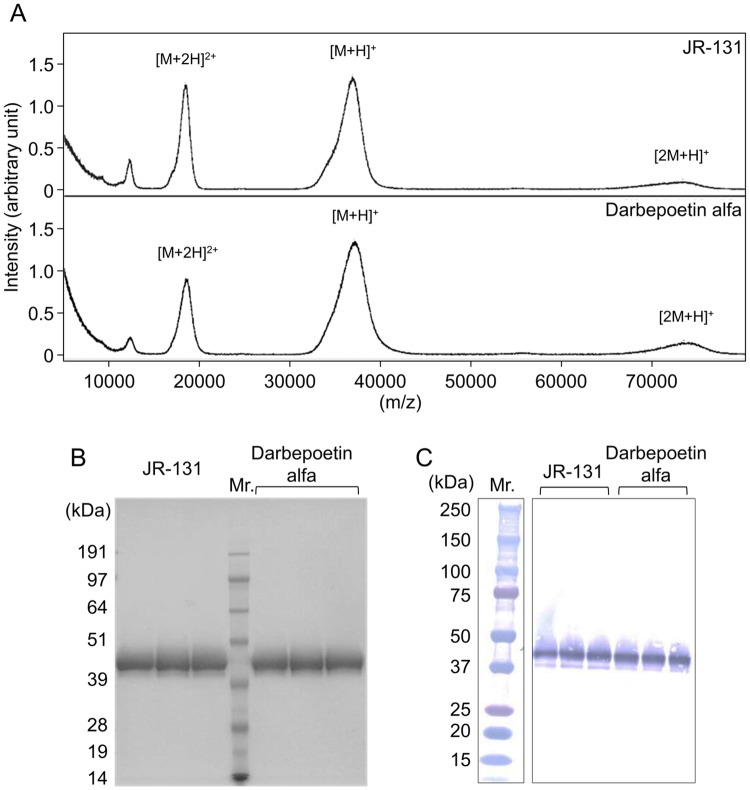
Molecular weight analyses of JR-131 and darbepoetin alfa. (A) MALDI-TOF/MS spectra of JR-131 (upper) and darbepoetin alfa (lower) are shown. (B) SDS-PAGE under reducing conditions showing JR-131 (left) and darbepoetin alfa (right) mobility. (C) Further immunoblot analysis using an anti-EPO antibody. Uncropped gel images are included in S1 Raw images.

The MALDI-TOF/MS spectra of JR-131 and darbepoetin alfa are shown in Fig 7A. The observed molecular weight of JR-131 was 36,000–37,000, which is similar to that of darbepoetin alfa. In addition, JR-131 and darbepoetin alfa showed the same mobility in SDS-PAGE (Fig 7B). In both samples, a higher-mobility band was detected just under the major band. Since the higher mobility band was detected by anti-human EPO antibody (Fig 7C), this band likely represents a low molecular weight form of the test substances, such as a carbohydrate-deficient form.

### *In vitro* efficacy assays

Binding affinity of the test substances for EPOR was evaluated using a surface plasmon resonance system with hEPOR-Fc fixed on Sensor Chip Protein A (S3 Fig). The K_D_ values of three different lots of JR-131 for hEPOR-Fc ranged from 0.79 to 0.83 × 10^−11^ mol/L. Comparatively, the K_D_ values of three different lots of darbepoetin alfa ranged from 0.79 to 0.86 × 10^−11^ mol/L (S3 Fig). These results demonstrate that the binding affinity of JR-131 for EPOR is similar to that of darbepoetin alfa.

**Fig 8:**
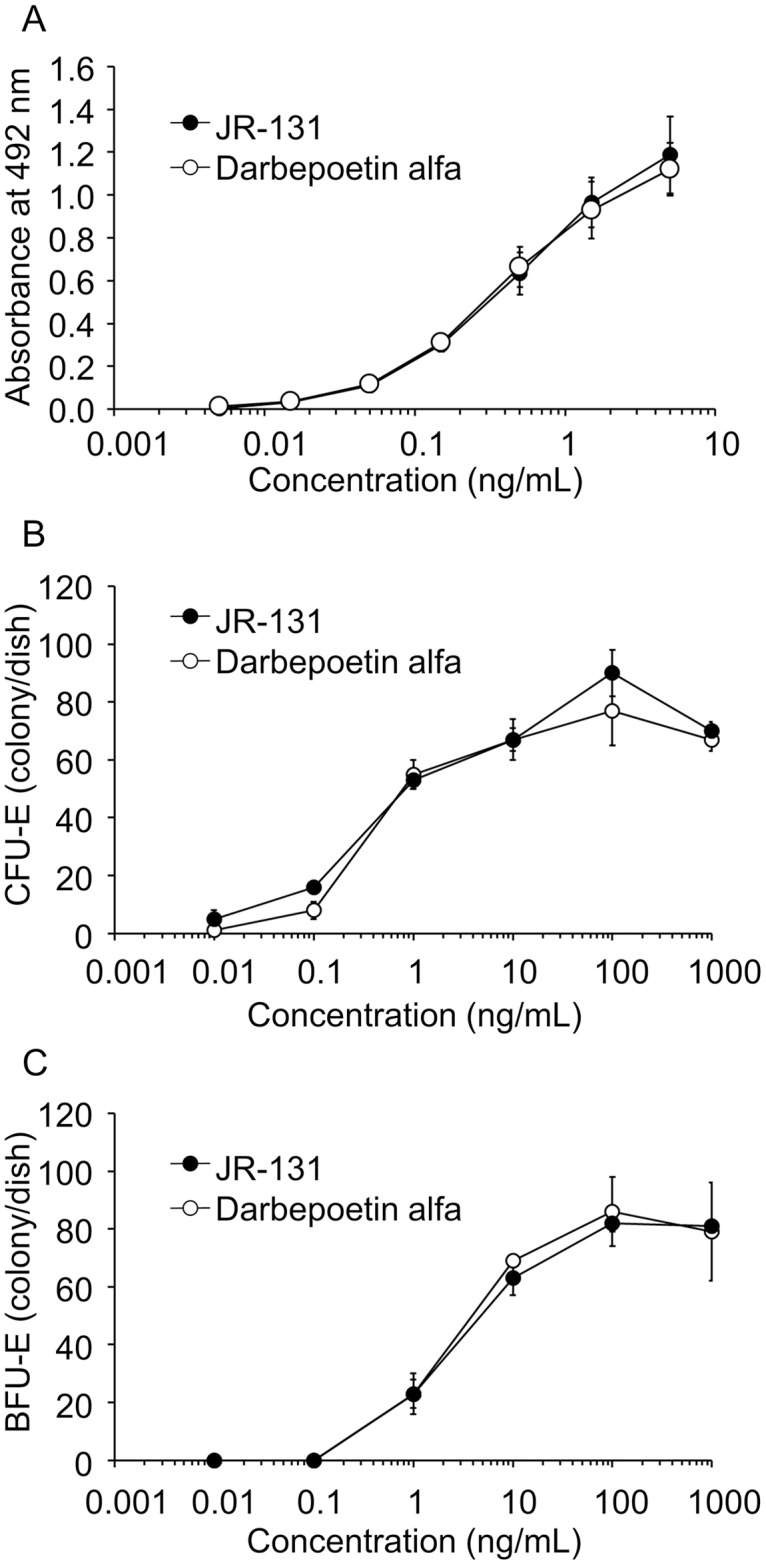
*In vitro* efficacy assays of JR-131 and darbepoetin alfa. (A) Agonist activity for EPOR was assessed by cell growth assay with BaF/EPOR cells. Biological activity was assessed by the effects of the test substances on the formation of (B) CFU-E- and (C) BFU-E-derived colonies using human bone marrow-derived mononuclear cells. Data are represented as means with standard deviation bars (n = 3).

Agonist activity of the test substances for EPOR was assessed using a cell growth assay with BaF/EPOR cells. The cells were confirmed to possess EPO-dependent proliferation and undergo apoptosis in the absence of EPO and IL-3. JR-131 facilitated the growth of BaF/EPOR cells in a concentration-dependent manner to a similar degree as did darbepoetin alfa (Fig 8A), indicating that the agonist activity for EPOR was not different between the two test substances.

*In vitro* biological activity was assessed by the effects of the test substances on the formation of CFU-E- and BFU-E-derived colonies using human bone-marrow-derived mononuclear cells. In the presence of either JR-131 or darbepoetin alfa, the number of both CFU-E- and BFU-E-derived colonies increased in a concentration-dependent manner within 0.01–100 ng/mL (Fig 8B and 8C). There were no differences in potency between the two test substances.

### *In vivo* efficacy assay in a rat model of renal anemia

**Fig 9:**
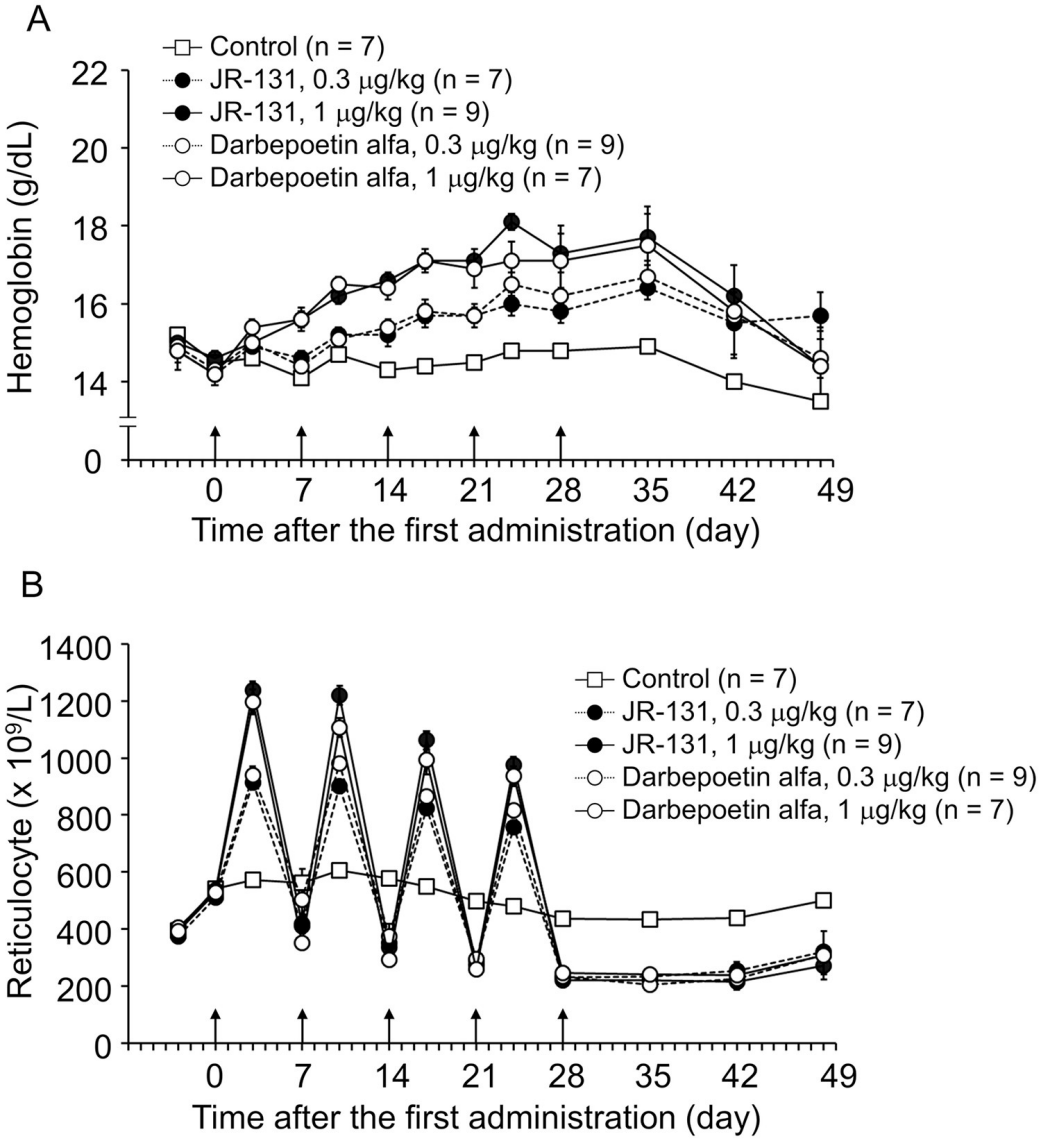
*In vivo* efficacy assay in 5/6 nephrectomized rats. The erythropoiesis-stimulating activity of the test substances was examined using an animal model of renal anemia. (A) Hemoglobin concentration and (B) reticulocyte number are shown. Arrows indicate the timing of drug administration. Data are represented as means with standard error bars. The numbers of animals are indicated in each graph.

The erythropoiesis-stimulating activity of the test substances was examined using an animal model of renal anemia [17]. JR-131 or darbepoetin alfa was administered intravenously at doses of 0.3 and 1 μg/kg to 5/6 nephrectomized rats. The hemoglobin concentration increased in all drug-treated groups and peaked at 24–35 days after the initial dosing, gradually decreasing thereafter (Fig 9A). The reticulocyte count increased in response to the dosing and decreased after withdrawal of the drugs in all drug-treated groups (Fig 9B). When comparing these effects between JR-131 and darbepoetin alfa at the same doses, no substantial differences were observed. These results indicate that the erythropoiesis-stimulating activity of JR-131 is comparable to that of its originator.

## Discussion

A biosimilar is a biological medicine comparable to an already approved biotechnological product in terms of quality, safety, and efficacy [18]. In the present study, we comprehensively characterized the physicochemical and biological properties of JR-131, a recombinant human protein produced using CHO cells as a host cell line, to demonstrate biosimilarity to darbepoetin alfa, an approved long-acting erythropoiesis-stimulating agent. In general, the difference in their glycan structures may well affect absorption and tissue distribution, resulting in different pharmacokinetics. Moreover, it is known that the activity of EPO is dependent on its glycan structure [19].

Our data showed that the degree of *O*-acetylation of sialic acid in JR-131 differs to some extent from that of darbepoetin alfa, which may affect *in vivo* clearance [16]. However, the pharmacokinetic parameters in humans including plasma t_1/2_ are similar for the two drugs [20], and the erythropoiesis-stimulating activity of these products in the model rats was not different (Fig 9), indicating that this glycan variation does not affect the pharmacokinetics and *in vivo* efficacy of the two drugs. In accordance with this finding, another proposed darbepoetin alfa biosimilar, LBDE, in which the *O*-acetylated sialic acid content is less than that of the originator, has pharmacokinetic profiles similar to those of darbepoetin alfa in rats [21].

The limitations of the present study include the lack of human studies. However, a randomized, double-blinded, parallel-group phase III study revealed JR-131 to have an equivalent therapeutic capability and similar safety profile to darbepoetin alfa [22]. In another phase III study, JR-131 maintained hemoglobin levels in patients with chronic kidney disease undergoing hemodialysis without clinically significant adverse events during the 52-week treatment [23]. Therefore, JR-131 proved to be a useful alternative to darbepoetin alfa in the management of renal anemia in patients undergoing hemodialysis. In conclusion, the present study provides a molecular and biological basis for JR-131 to act as a biosimilar of darbepoetin alfa.

## Supporting information

S1 Fig Amino acid sequence of JR-131. (PDF)

S2 Fig Mirror images of deconvoluted mass spectra of glycopeptides derived from JR-131 (upper) and darbepoetin alfa (lower). Mass spectra of glycopeptides containing Asn24 and Asn30 (A), glycopeptides containing Asn38, Asn83, and Asn88 (B), and glycopeptides containing Asn38 (C) are shown.
(PDF)

S3 Fig Binding affinity of JR-131 and darbepoetin alfa for EPOR. Upper graphs are representative sensorgrams of Surface Plasmon Response. Values of K_D_, k_a_, and k_d_ are also shown.
(PDF)

S1 Raw images Uncropped gel images for Fig 7B and 7C. (PDF)

S1 Checklist The ARRIVE guidelines checklist. (PDF)
