# Supplementary material for: Physicochemical and biological evaluation of JR-131 as a biosimilar to a long-acting erythropoiesis-stimulating agent darbepoetin alfa
Source: PLoS One. 2020 Apr 17;15(4):e0231830. doi: 10.1371/journal.pone.0231830 (PMC7164597; doi:10.1371/journal.pone.0231830)
Supplement: S1 Fig — (PDF) [file pone.0231830.s001.pdf]

APPRLICDSR VLERYLLEAK EAENITTGCN ETCSLNENIT VPDTKVNFYA
WKRMEVGQQA VEVWQGLALL SEAVLRGQAL LVNSSQVNET LQLHVDKAVS
GLRSLTTLLR ALGAQKEAIS PPDAASAAPL RTITADTFRK LFRVYSNFLR
GKLKLYTGEA CRTGD

C-C : disulfide bond
Underline : glycosylation site

S1 Fig. Amino acid sequence of JR-131.
