# Supplementary material for: Physicochemical and biological evaluation of JR-131 as a biosimilar to a long-acting erythropoiesis-stimulating agent darbepoetin alfa
Source: PLoS One. 2020 Apr 17;15(4):e0231830. doi: 10.1371/journal.pone.0231830 (PMC7164597; doi:10.1371/journal.pone.0231830)
Supplement: S2 Fig — Mass spectra of glycopeptides containing Asn24 and Asn30 (A), glycopeptides containing Asn38, Asn83, and Asn88 (B), and glycopeptides containing Asn38 (C) are shown. (PDF) [file pone.0231830.s002.pdf]

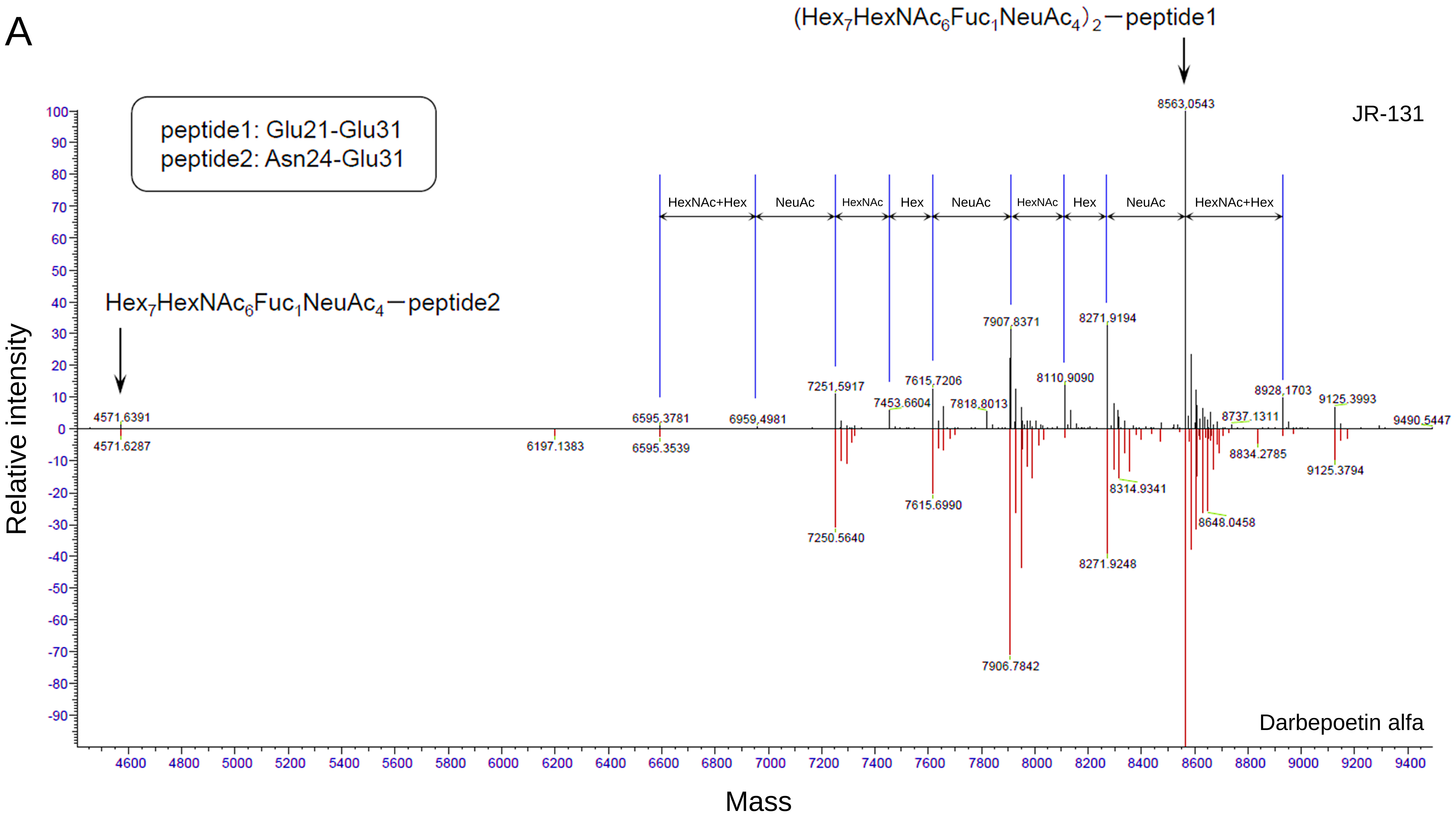

S2A Fig. Mirror images of deconvoluted mass spectra of glycopeptides derived from JR-131 (upper) and darbepoetin alfa (lower). Mass spectra of glycopeptides containing Asn24 and Asn30 are shown.

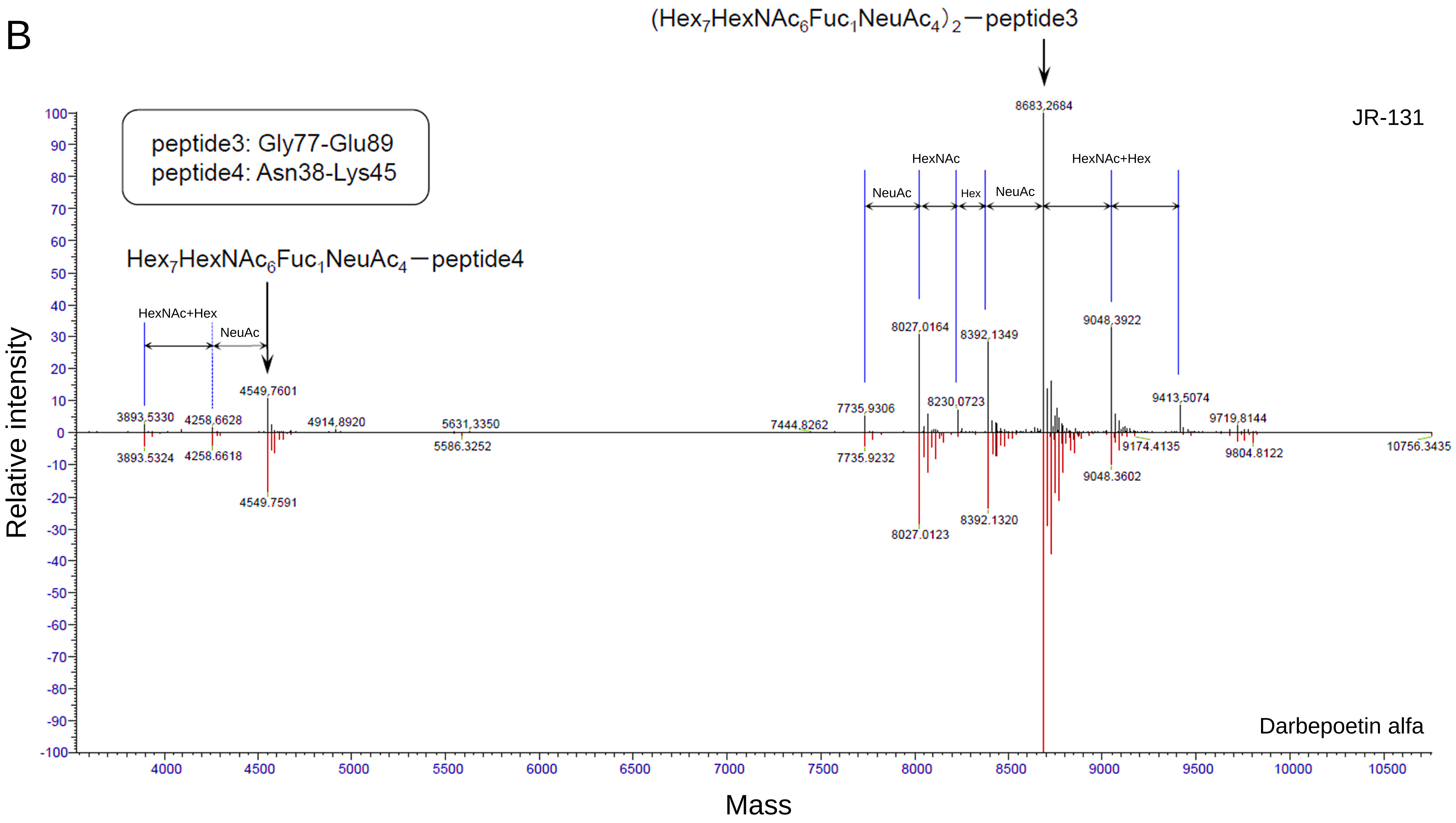

S2B Fig. Mirror images of deconvoluted mass spectra of glycopeptides derived from JR-131 (upper) and darbepoetin alfa (lower). Mass spectra of glycopeptides containing Asn38, Asn83 and Asn88 are shown.

C

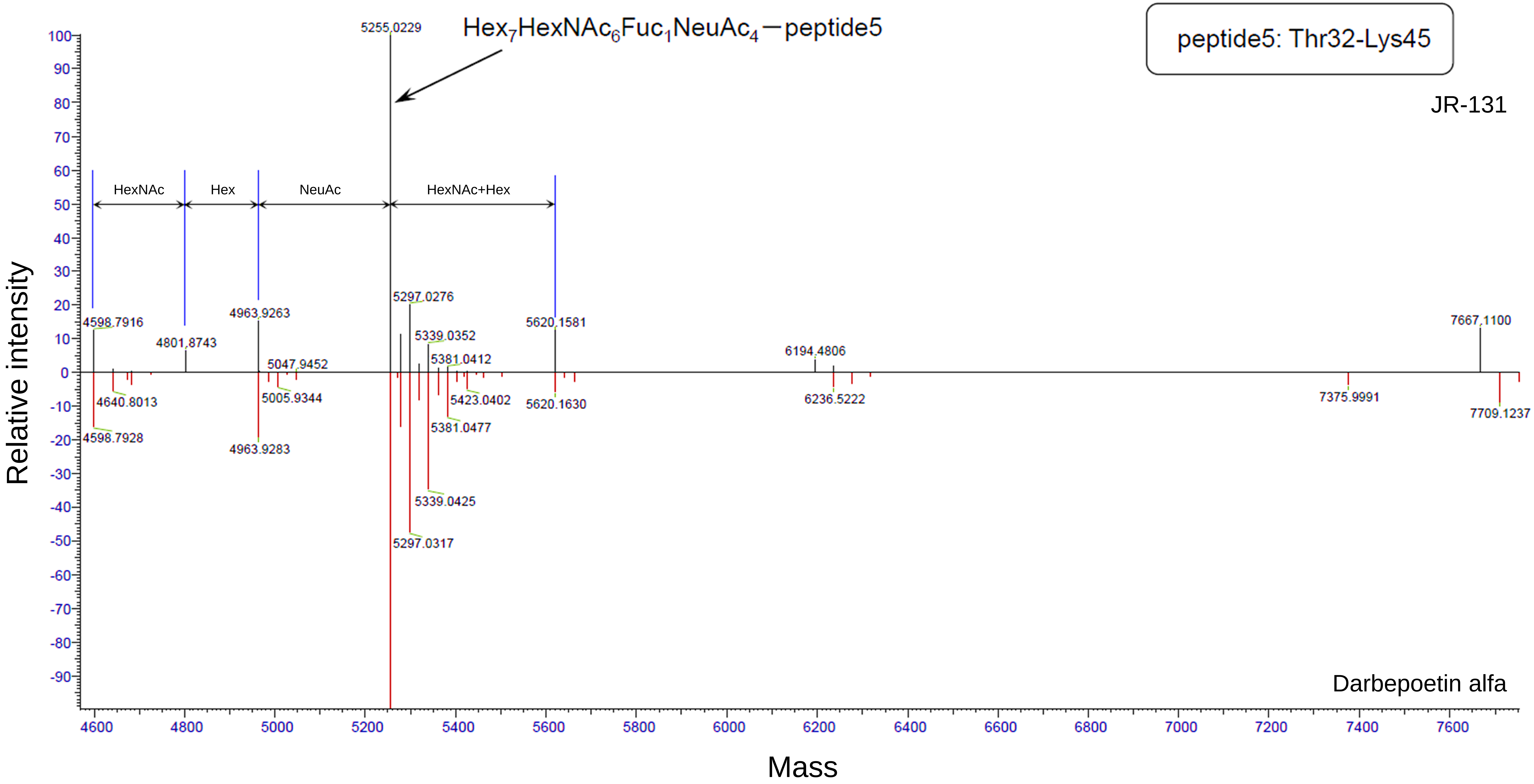

S2C Fig. Mirror images of deconvoluted mass spectra of glycopeptides derived from JR-131 (upper) and darbepoetin alfa (lower). Mass spectra of glycopeptides containing Asn38 are shown.
