# Supplementary material for: Physicochemical and biological evaluation of JR-131 as a biosimilar to a long-acting erythropoiesis-stimulating agent darbepoetin alfa
Source: PLoS One. 2020 Apr 17;15(4):e0231830. doi: 10.1371/journal.pone.0231830 (PMC7164597; doi:10.1371/journal.pone.0231830)
Supplement: S3 Fig — Upper graphs are representative sensorgrams of Surface Plasmon Response. Values of KD, ka, and kd are also shown. (PDF) [file pone.0231830.s003.pdf]

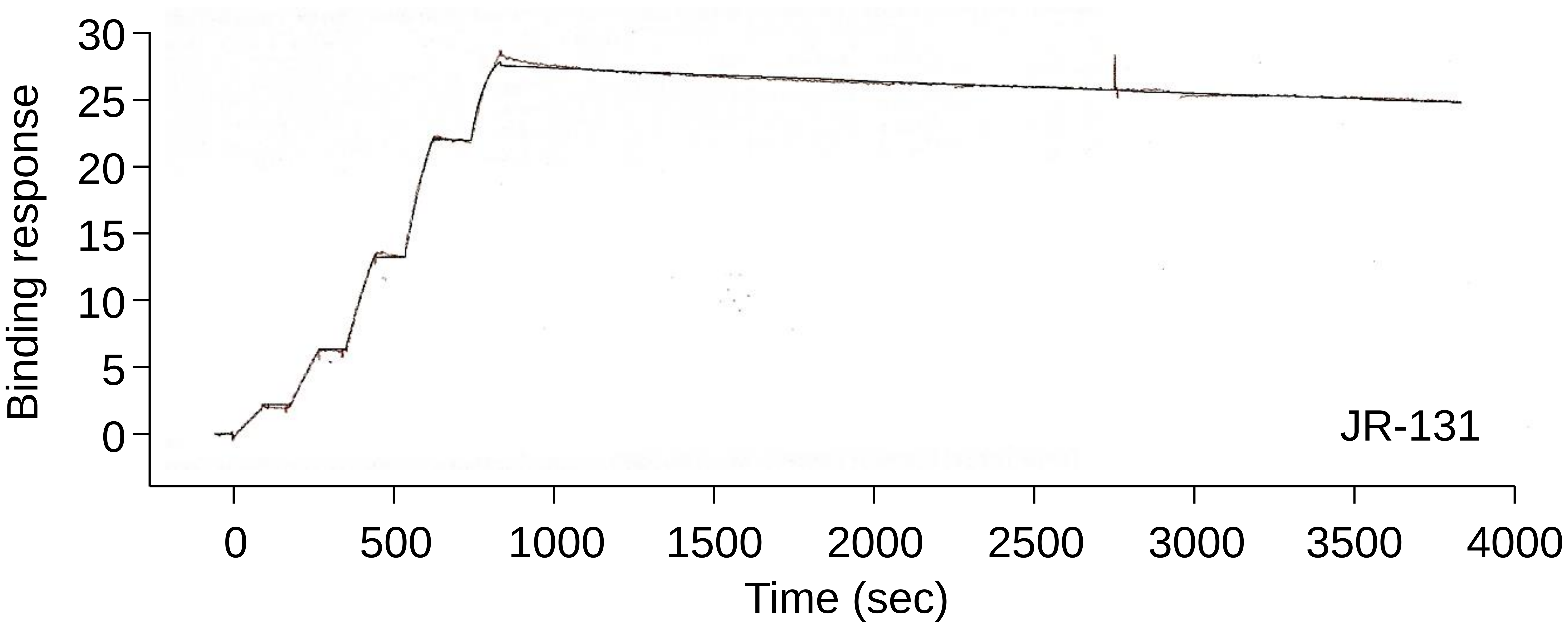

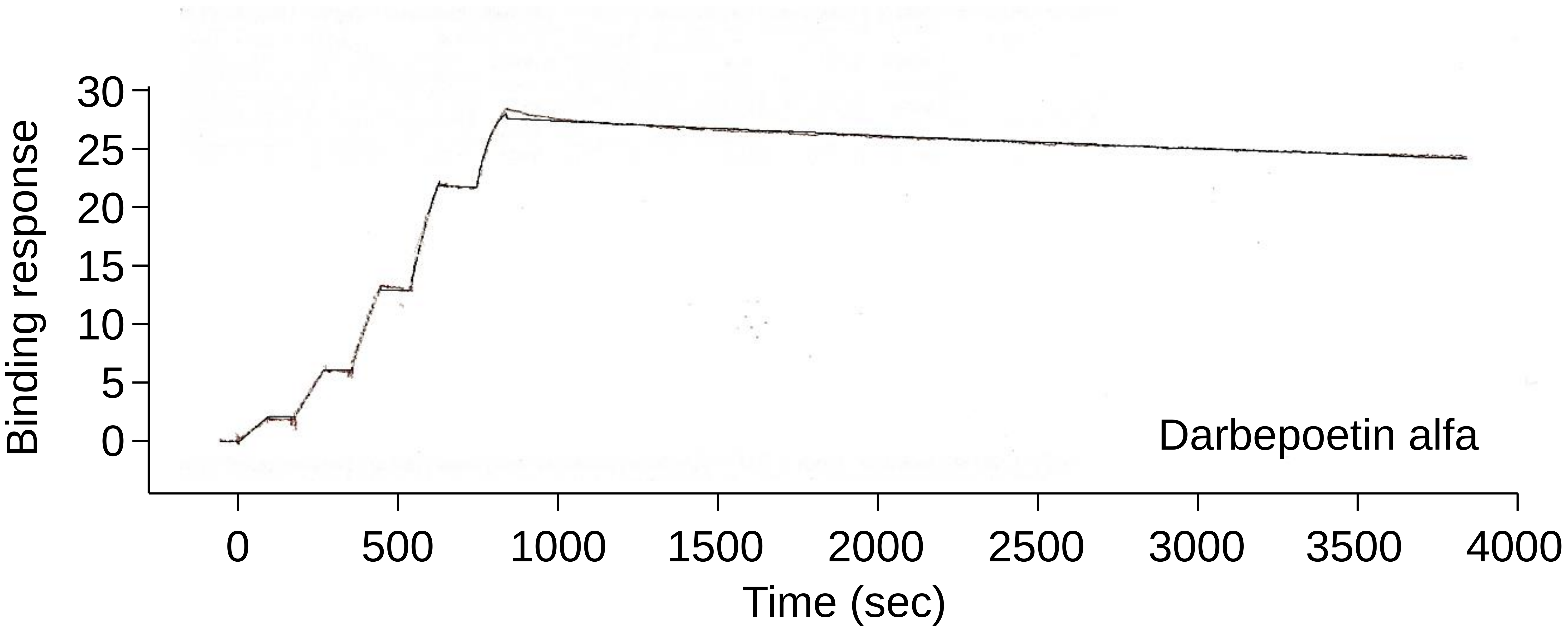

		k_a ($\times 10^6$ $M^{-1}\cdot S^{-1}$)	k_d ($\times 10^{-5}$ S^{-1})	K_D ($\times 10^{-11}$ M)
JR-131	lot 1	6.21	4.93	0.79
	lot 2	6.15	4.90	0.80
	lot 3	6.09	5.07	0.83
Darbepoetin alfa	lot 1	6.07	5.05	0.84
	lot 2	5.78	4.97	0.86
	lot 3	6.20	4.91	0.79

S3 Fig. Binding affinity of JR-131 and darbepoetin alfa for EPOR. Upper graphs indicate representative sensorgrams of Surface Plasmon Response. Values of K_D, k_a, and k_d are also shown.
