# Supplementary material for: Physicochemical and biological evaluation of JR-131 as a biosimilar to a long-acting erythropoiesis-stimulating agent darbepoetin alfa
Source: PLoS One. 2020 Apr 17;15(4):e0231830. doi: 10.1371/journal.pone.0231830 (PMC7164597; doi:10.1371/journal.pone.0231830)
Supplement: S1 Raw images — (PDF) [file pone.0231830.s004.pdf]

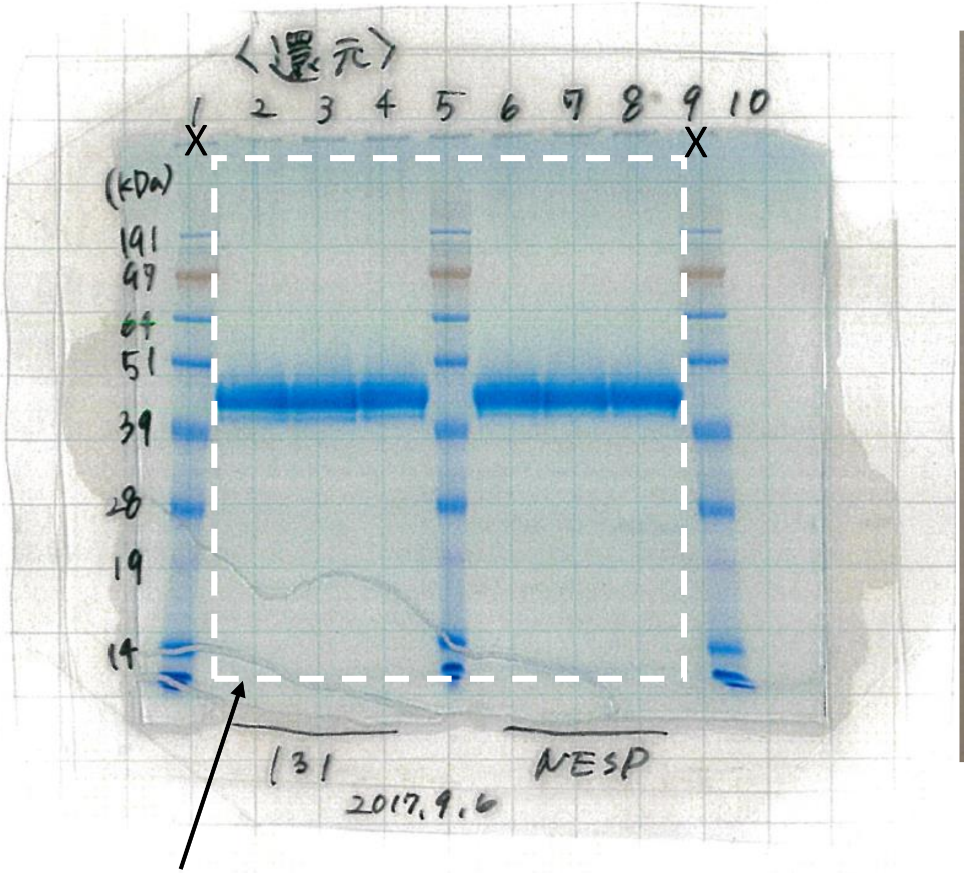

Cropped area for Fig 7B

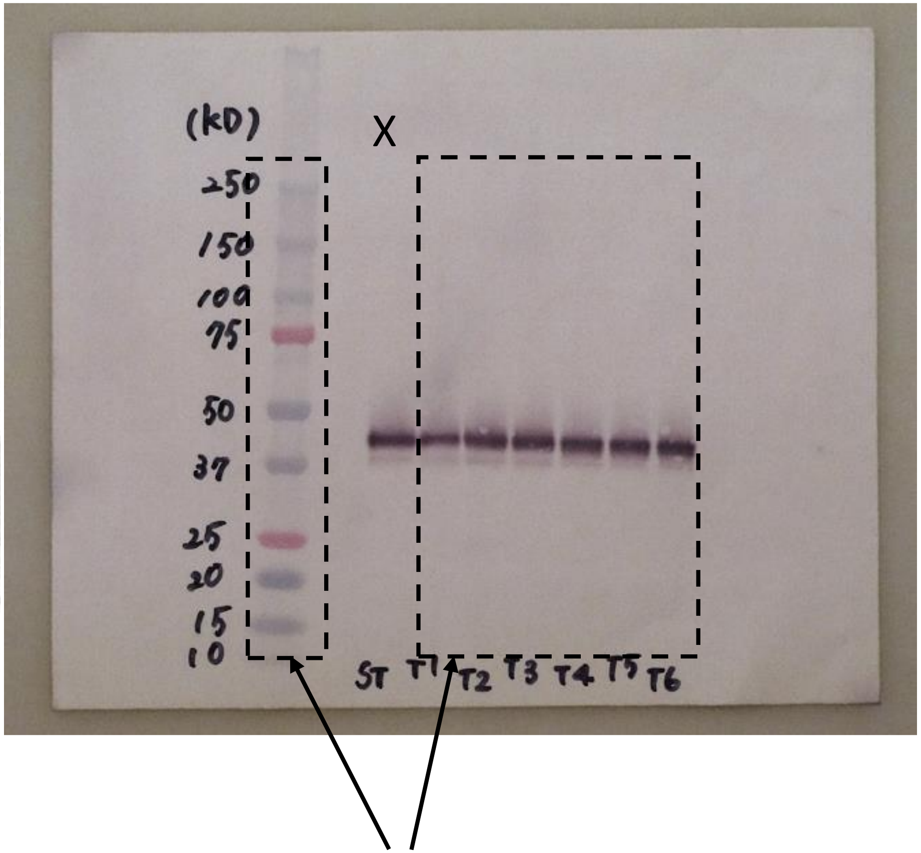

Cropped area for Fig 7C

S1_raw_images. Uncropped gel images for Fig 7B and C.
Gel images were obtained by an image scanner.
